# Objective and automatic grading system of facial signs from selfie pictures of South African women: Characterization of changes with age and sun‐exposures

**DOI:** 10.1111/srt.13153

**Published:** 2022-05-01

**Authors:** Frederic Flament, Yuze Zhang, Ruowei Jiang, Cecilia Trehin, Matthieu Cassier, Caroline Delaunay, Guive Balooch, Camille Kroely

**Affiliations:** ^1^ L'Oréal Research and Innovation Clichy France; ^2^ ModiFace–A L'Oréal Group Company Toronto Ontario Canada; ^3^ Eurosyn Villebon‐sur‐Yvette France; ^4^ L'Oréal CDO–Digital Service Factory Clichy France

**Keywords:** artificial intelligence, automatic grading system, facial signs, photo‐aging, South African women

## Abstract

**Objective:**

To evaluate the capacity of the automatic detection system to accurately grade, from smartphones’ selfie pictures, the severity of fifteen facial signs in South African women and their changes related to age and sun‐exposure habits.

**Methods:**

A two‐steps approach was conducted based on self‐taken selfie images. At first, to assess on 306 South African women (20–69 years) enrolled in Pretoria area (25.74°S, 28.22°E), age changes on fifteen facial signs measured by an artificial intelligence (AI)‐based automatic grading system previously validated by experts/dermatologists. Second, as these South African panelists were recruited according to their usual behavior toward sun‐exposure, that is, nonsun‐phobic (NSP, *N* = 151) and sun‐phobic (SP, *N* = 155) and through their regular and early use of a photo‐protective product, to characterize the facial photo‐damages.

**Results:**

(1) The automatic scores showed significant changes with age, by decade, of sagging and wrinkles/texture (*p* < 0.05) after 20 and 30 years, respectively. Pigmentation cluster scores presented no significant changes with age whereas cheek skin pores enlarged at a low extent with two plateaus at thirties and fifties. (2) After 60 years, a significantly increased severity of wrinkles/texture and sagging was observed in NSP versus SP women (*p* < 0.05). A trend of an increased pigmentation of the eye contour (*p* = 0.06) was observed after 50 years.

**Conclusion:**

This work illustrates specific impacts of aging and sun‐exposures on facial signs of South African women, when compared to previous experiments conducted in Europe or East Asia. Results significantly confirm the importance of sun‐avoidance coupled with photo‐protective measures to avoid long‐term skin damages. In inclusive epidemiological studies that aim at investigating large human panels in very different contexts, the AI‐based system offers a fast, affordable and confidential approach in the detection and quantification of facial signs and their dependency with ages, environments, and lifestyles.

## 1 INTRODUCTION

The efficacy of cosmetic products can be rationally assessed in vivo under three complementary approaches: instrumentally (when possible), clinically and responses from the human panel under test. A good coherence between these three domains thus confers to the product efficacy a strong relevance. This paradigm seems however entering into a new era that concerns both consumers and/or clinical studies as the consumer under test becomes prone at supplying and checking objective data. Such is the recent case of automatic grading system for detection and quantification of facial signs from selfie pictures, thanks to smartphones with powerful hardware and high‐resolution cameras.

As for now, mostly focusing on facial images, these allow to rapidly obtain digital data on large cohorts, in specific environments or lifestyles. Hence, these new embarked automatic grading systems that detect and quantify of facial signs are powerful methodologies to better depict and quantify the slow process of facial skin aging, under real‐life conditions, on large panels of humans of various ages, backgrounds and habits.

These systems basically ground on artificial intelligence (AI) and deep‐learning‐based algorithms dedicated to medical purposes^1^, ^2^, ^3^ or cosmetical applications. As examples, an AI‐based automatic grading system^4^, ^5^ allowed to attribute the grading of the severity of facial signs using skin aging atlases^6^, ^7^ taken as a reference, on differently aged women with dark skin tones. This algorithm has been trained and validated versus scoring obtained from experts and dermatologists.

This system was applied to changes in skin color, related to age, geographical locations, and environmental conditions, that is, the so‐called exposome.^8^ Of note, skin aging leads to cultural impacts as some facial signs own different weights in the overall perception of a younger or older aspect, as demonstrated in different locations such as South Africa.^9^, ^10^ However, with regard to constitutive dark skin tones, the relative impacts of photo‐aging^11^ and photo‐protection,^12^, ^13^, ^14^ especially in African people, remain an open field of research, with clear specificities.^15^, ^16^, ^17^ Same holds true with the still fuzzy characterization of intrinsic and extrinsic factors of skin aging in these dark skin tones. In addition, the individual relationship with sun‐exposure (sun‐phobic or nonsun phobic) plays a crucial role in the development of facial aging, as shown by clinical studies in various countries such as France,^18^ China,^19^ and Japan.^20^, ^21^

Accordingly, as new approach, the AI‐based grading system was applied to differentiate the impacts on facial aging by sun‐phobic and nonsun‐phobic behaviors on 306 differently aged South Africa women living in Pretoria (25.74°S, 28.22°E), using images collection and automatic quantification from selfie pictures. The results of this study are the objects of the present paper.

## 2 MATERIAL AND METHODS

### 2.1 Subjects and conditions of privacy in the collection of smartphones’ selfie pictures

Three hundred six South African women of different ages (20−69 years) with photo‐types V and VI were recruited in Pretoria (25.74°S, 28.22°E), where all permanently reside since more than 15 years. Based on the clustering method described above–already published^18^, ^19^, ^20^, ^21^–two groups were identified according to their own sun‐exposures habits, that is, sun‐phobic (SP) and nonsun‐phobic (NSP).

### 2.2 Clustering procedure for sun‐phobic and nonsun‐phobic groups

To establish their usual relationships with sun‐exposures, subjects were asked to fill a dedicated questionnaire (Table 1). The latter aimed at establishing a sun history and habits index (SHHI) by taking into account their daily sun‐exposures (>4 h a day) and the regular use (or not) of a photo‐protective cosmetic product. An index is obtained by multiplying, in each age‐range, the numbers (1, 2, or 3) of the exposure by those (1 or 2) corresponding to the use of a photo‐protective product. The global SHHI is obtained by adding all four indexes, further divided by 4. Hence, this index varies from 1 to 6, where 1 corresponds to the least degree of sun‐exposures ([1 + 1 + 1 + 1]/4) and 6 ([6 + 6 + 6 + 6]/4) as the most extreme case being a regular sun‐exposure at young ages, devoid of photo‐protection. In such calculated index, value of 3.5 represents women who are regularly exposed to sun with a systematic photo‐protective habit, that is, the intermediate case.

**TABLE 1:** Questionnaire used to evaluate the history of sun‐exposures (combined or not to a photo‐protection habit) of the South African women enrolled in the study. In bold, the numbers used for calculating the sun history and habits index (SHHI)

Have you ever spent more than 4 h a day outdoors for professional or recreational activities			
Between 0 and 7 years old…	**1–**No	**2–**Occasionally	**3–**Regularly
with sun protection?	**1–**Yes	**2–**No	
Between 7 and 18 years old…	**1–**No	**2–**Occasionally	**3–**Regularly
with sun protection?	**1–**Yes	**2–**No	
After 18 years old…	**1–**No	**2–**Occasionally	**3–**Regularly
with sun protection?	**1–**Yes	**2–**No	
At the present time…	**1–**No	**2–**Occasionally	**3–**Regularly
with sun protection?	**1–**Yes	**2–**No	

Hence, the threshold between SP and NSP subjects was arbitrarily set at 3.5. These clustering criteria led to the creation of two groups, that is, sun‐phobic (*N* = 155) and nonsun‐phobic subjects (*N* = 151) women, well balanced in ages and SHHI–see above. Table 2 gathers other questions related to “exposome”^8^ to establish equivalence among the two groups on all other factors, behavior toward sun excepted.

**TABLE 2:** Questionnaire used to evaluate the equivalence of two cohorts created with sun history and habits index (SHHI) on other internal and external exposome factors for the Japanese women enrolled in the South African study

Equivalence of cohorts versus exposome factors					
Do you smoke? (cigarettes/day)	1–No	2–1 to 5	3–6 to 10	4–11 to 20	5–More than 20
Do you drink alcohol? (glasses/day)	1–No	2–1	3–2 to 5	4–More than 5	N/A
How much time do you spend in transport to go to work or other activities (round‐trip), per day?	1–Less than 1 h	2–1 to 2 h	3–2 to 3 h	4–3 to 4 h	5–More than 4 h

Abbreviation: N/A, not applicable.

### 2.3 Protocol

The protocol was tiered into three successive steps:

Step 1: All 306 subjects were asked to take one selfie image (full face, frontal camera of smartphone) at morning, under the conditions exposed above.
Step 2: Selfie images were analyzed by the automatic grading system algorithm,^4^, ^5^ and the resulting scores of the 15 facial signs (Table 3) were sent to our secured website under blind codes.
Step 3: Automatic scores were analyzed to characterize age‐related changes or sun‐exposures habits impacts.

**TABLE 3:** Fifteen clinical facial signs and their respective photographic scale's ranges for African women used in artificial intelligence (AI)‐based automatic grading system. They cover different clinical clusters: wrinkles/texture, sagging, pigmentation signs, and cheek pores

Clinical clusters	Facial signs	Definition	Asian scales
Wrinkles/Texture (eight signs)	Forehead wrinkles	Depth of the transverse wrinkles on the forehead.	0−6
	Glabellar wrinkles	Depth of vertical wrinkles between eyebrows.	0−7
	Periorbital wrinkles (upper cheek area)	Depth of folds at malar area below Crow's feet, eye orbit excepted.	0−4
	Nasolabial fold	Depth of the fold present between the base of the nose and lips.	0−6
	Marionette lines	Depth of folds at the corner of lips.	0−5
	Cheek folds	Depth of folds in cheek area.	0−6
	Texture of the mouth contour	Thick and cracked appearance of the mouth region (solar elastosis).	0−7
	Crow's feet wrinkles	Depth of wrinkles at the area of outer eye corner.	0−4
Pigmentation signs (four signs)	Density of pigmentary spots	Number of spots per area unit on the cheek.	0−6
	Pigmentation of the eye contour	Intensity and surface covered by pigmentation and surrounding eye.	0−4
	Pigmentary exgrowth	Dermatosis papulosis nigra. Number and severity of pigmentary exgrowth.	0−6
	Depigmented area of skin surface	Surface of facial skin presenting hypo‐pigmentation.	0−5
Ptosis/Sagging (two signs)	Ptosis of lower part of the face	Sagging severity of the lower parts each side of the chin.	0−6
	Eye bags	Intensity of the ocular bulge.	0−5
Cheek pores (one sign)	Cheek skin pores	Size of visible pores on the cheek irrespective of their densities.	0−5

### 2.4 Facial signs assessed by dermatologists and by automatic grading system

The interest of using standard photographic scales to bring reliability and robustness in clinical evaluation of darkest skin tones was previously discussed.^22^, ^23^, ^24^ Table 3 illustrates the 15 facial signs (definitions and respective scoring ranges) that were further analyzed by the AI‐based automatic grading system. The latter, based on smartphones’ selfie images on South African subjects, uses the standardized grades of the skin aging atlases as reference.^6^, ^7^ For sake of clarity, clinical results could be gathered in facial clinical clusters, obtained by arithmetically transforming the respective grades of the eight signs of wrinkles/texture (0−4 to 0−7), the four signs of pigmentation (0−4 to 0−6), the two signs of sagging (0−5, 0−6), and the one sign of cheek pores (0−5) into a same 0−5 scale for being averaged.

The training of the AI‐based automatic grading system dedicated to women with darkest skin tones and its validation versus experts and dermatologists has been previously described.^4^, ^5^ In short, it is a supervised regression problem within deep learning framework based on convolution neural networks.

### 2.5 General characteristics of the cohorts and conditions of privacy in the collection of smartphones’ selfie pictures

Table 4 summarizes the distribution of subjects by age‐classes and sun‐exposures habits.

**TABLE 4:** Distribution of the 306 studied South African women according to age‐classes and sun‐exposure behaviors

Panels/Age clusters	Total population	SP	NSP
20−29 years	55	30	25
30−39 years	93	47	46
40−49 years	44	25	19
50−59 years	64	25	39
60−69 years	50	28	22
Total	306	155	151

Abbreviations: NSP, nonsun‐phobic; SP, sun‐phobic.

The two groups were at best balanced in ages‐classes following some imperative inclusion criteria (Table 4). These were as follows: (1) possessing a smartphone (any brand) with a high‐resolution camera (≥5 Megapixels), (2) used to take selfie pictures, and (3) free from any facial skin disease, disorder or scars (rosacea, acne, angioma, etc). All subjects were fully informed about the objective of the study and signed an informed consent. The latter guaranteed that their photographs were totally confidential (blind‐coded) and further deleted once analyzed.

**FIGURE 1:**
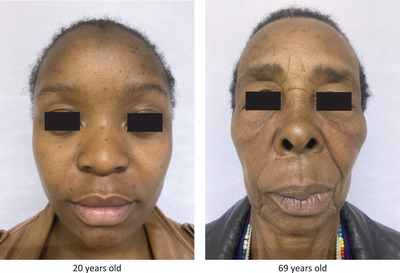
Two illustrative smartphone's images of two South African women enrolled in the study

Subjects were asked to take frontal selfie pictures–examples displayed in Figure 1–, under indoor artificial diffused lighting condition, at morning, their faces being free from any cosmetic adornment (i.e., bare skin), and to adopt the most neutral expression in the shooting. Some examples of exploitable selfie pictures were provided to all as a help for taking the most accurate image (distance and angle) stored by the smartphone. All pictures were then sent to our dedicated and secured website for being further processed.

### 2.6 Statistics

1. The gradings provided by the automatic grading system for all 306 subjects were analyzed using an ANOVA test (SPSS software Package, Chicago, ILL, USA) according to two factors (automatic scores, ages). The Newman–Keuls test (95% confidence interval) was used to analyze the significance of all differential values in each age‐class and for each facial sign.
2. Aiming at comparing the two groups of subjects (e.g. SP vs. NSP), for two age‐classes and fifteen facial clinical signs, a comparison of their respective average scores was calculated, using the Student *t*‐test, taking a *p* < 0.05 as significant threshold for independent samples.

## 3 RESULTS

### 3.1 Facial signs computed by AI‐based automatic grading system and age

**FIGURE 2:**
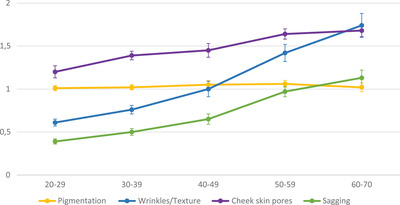
Mean ± CI for clinical clusters based on facial signs evaluated on selfie pictures by artificial intelligence (AI)‐based automatic grading system and their changes with age observed in the cohort of South African. CI, Confidence Interval (95%)

Table 5 illustrates the progressive changes of the 15 facial signs, and Figure 2 shows the four clinical‐associated clusters (mean grades ± confidence interval) with age. Globally, the changes on lifespan remain low in term of grading units compared to other populations.^25^ Wrinkles/Texture and sagging appear of a rather regular rate (*p* < 0.05) after 20 and 30 years respectively whereas pigmentation shows no changes with age. Surprisingly, individual pigmentation signs evaluated by AI‐based automatic grading system show erratic changes among nature of disorders, that is, no changes for pigmentary exgrowth or in depigmented surface, decreased density of dark spots and increase in the pigmentation of the eye contour. Finally, cheek skin pores present a rather low rate of enlargement with age somewhat plateauing at thirties and fifties (*p* < 0.05).

**TABLE 5:** Changes of 15 facial signs with ages in 306 South African women (Mean ± confidence interval 95% [CI]) measured by artificial intelligence (AI)‐based automatic grading system. Green or grey colors cells correspond to nonsignificant differences between them. Each white cell is significantly different from all others

Age‐clusters/Facial signs	20−29 years	30−39 years	40−49 years	50−59 years	60−69 years
Forehead wrinkles	0.55 ± 0.12	0.69 ± 0.10	0.99 ± 0.22	1.58 ± 0.23	2.14 ± 0.28
Glabellar wrinkles	0.44 ± 0.05	0.61 ± 0.08	0.79 ± 0.12	1.28 ± 0.20	1.96 ± 0.34
Periorbital wrinkles (upper cheek area)	1.08 ± 0.08	1.20 ± 0.07	1.45 ± 0.12	1.70 ± 0.11	1.88 ± 0.16
Nasolabial fold	0.99 ± 0.07	1.30 ± 0.11	1.79 ± 0.24	2.67 ± 0.21	2.97 ± 0.24
Marionette lines	0.82 ± 0.06	0.92 ± 0.07	1.13 ± 0.12	1.42 ± 0.09	1.67 ± 0.12
Cheek folds	0.39 ± 0.03	0.54 ± 0.04	0.71 ± 0.07	1.02 ± 0.07	1.24 ± 0.10
Texture of the mouth contour	0.25 ± 0.03	0.35 ± 0.03	0.5 ± 0.07	0.87 ± 0.09	1.15 ± 0.14
Crow's feet wrinkles	0.45 ± 0.04	0.61 ± 0.05	0.85 ± 0.10	1.39 ± 0.12	1.74 ± 0.16
Density of pigmentary spots	2.30 ± 0.11	2.19 ± 0.07	2.07 ± 0.11	2.09 ± 0.09	2.03 ± 0.11
Pigmentation of the eye contour	1.25 ± 0.08	1.29 ± 0.06	1.46 ± 0.11	1.47 ± 0.10	1.39 ± 0.10
Pigmentary exgrowth	0.41 ± 0.04	0.48 ± 0.04	0.49 ± 0.08	0.49 ± 0.06	0.48 ± 0.06
Depigmented area of skin surface	0.23 ± 0.03	0.26 ± 0.02	0.26 ± 0.03	0.26 ± 0.03	0.26 ± 0.04
Ptosis of lower part of the face	0.62 ± 0.04	0.78 ± 0.06	1.04 ± 0.10	1.54 ± 0.10	1.80 ± 0.15
Eye bags	0.26 ± 0.03	0.35 ± 0.04	0.43 ± 0.06	0.66 ± 0.07	0.75 ± 0.08
Cheek skin pores	1.20 ± 0.07	1.39 ± 0.05	1.45 ± 0.08	1.64 ± 0.06	1.68 ± 0.07

Abbreviation: CI, confidence interval 95%.

### 3.2 Sun‐exposures impact on facial signs

Table 6 gathers the significant and relevant differences among SP and NSP groups for the fifteen facial signs assessed by the AI‐based automatic grading system according to age‐classes. The impacts of sun‐exposures on South African panelists were significantly found after 60 years old. These lead to significant and large increase in wrinkles/texture signs (nasolabial fold, marionette lines, cheek folds, texture of mouth contour) and sagging (ptosis of the lower part of the face). Interestingly, in the fifties, despite rather low amplitude of changes in pigmentated signs, close to being significant, an impact of sun‐exposures in the pigmentation of the eye contour was detected.

**TABLE 6:** Changes in the 15 facial signs scoring observed between sun‐phobic (*N* = 155) and nonsun‐phobic (*N* = 151) behaviors for each age‐class. Differences between these values represent the impact of sun exposures upon clinical signs severity. In regular black, those where differences did not statistically differ. In bold green, all signs that present a significant (*p* < 0.05) negative difference for nonsun‐phobic (NSP), as compared to SP. In bold red, all signs that present a significant (*p* < 0.05) positive difference ≥0.20 grade for NSP, as compared to SP. In regular red, all signs that present a limit significant (*p* ≤ 0.10) positive difference ≥0.15 grade for NSP, as compared to SP. L, limit significant; NS, not significant; S, significant

Age‐clusters/Facial signs	20−29 years	30−39 years	40−49 years	50−59 years	60−69 years
Forehead wrinkles	NS	NS	NS	NS	NS
Glabellar wrinkles	NS	NS	NS	NS	NS
Periorbital wrinkles	NS	NS	NS	NS	NS
Nasolabial fold	NS	NS	NS	NS	**S (*p* < 0.05)** **Δ (NSP‐SP) = 0.49**
Marionette lines	NS	NS	NS	NS	**L (*p* = 0.10)** **Δ (NSP‐SP) = 0.20**
Cheek folds	NS	NS	NS	NS	L (*p* = 0.10) Δ (NSP‐SP) = 0.16
Texture of the mouth contour	NS	NS	NS	NS	**S (*p* < 0.05)** **Δ (NSP‐SP) = 0.49**
Crow's feet wrinkles	NS	NS	NS	NS	NS
Density of pigmentary spots	NS	NS	NS	NS	NS
Pigmentation of the eye contour	NS	NS	NS	L (*p* = 0.06) Δ (NSP‐SP) = 0.20	NS
Pigmentary exgrowth	NS	**S (*p* < 0.05)** **Δ (NSP‐SP) = −0.13**	NS	NS	NS
Depigmented area of skin surface	NS	NS	NS	NS	NS
Ptosis of lower part of the face	NS	NS	NS	NS	**S (*p* < 0.05)** **Δ (NSP‐SP) = 0.28**
Eye bags	NS	NS	NS	NS	NS
Cheek skin pores	NS	NS	NS	NS	NS

### 3.3 Answers from questionnaire

Attempts to differentiate the SP and NSP groups through their conditions and duration of transportation and/or their alcohol and smoking habits (Table 2) failed. Such negative findings suggest that the significant differences, obtained by AI‐based automatic grading system analysis are, in a large part, most linked to the different sun‐exposure habits.

## 4 DISCUSSION

The methodology using selfie images coupled to AI‐based automatic grading is a promising approach, previously validated versus experts and dermatologists gradings on Asian, African, and Caucasian skins.^4^, ^5^ The latter study^5^ indeed showed significant agreements between experts and automatic gradings of all facial signs (wrinkles/skin texture, sagging, cheek pores etc.) although less significant in pigmentation signs of dark skin complexions where the contrast between a small pigmented spot and its surrounding dark background is low, a classic source of variable assessments by both human eyes and optical recording systems.

The present study globally found a lower dynamic of facial skin aging in African skin than that of other ethnicities,^25^ that is, showing delayed manifestations of aging.^26^ As previously published,^6^, ^7^, ^27^, ^28^ the early onset (mean age) of visible wrinkles (grade > 1) much varies among populations with 33 years for European, 39 years for East Asian, 40 years for Indian, and 52 years for African descent women.

These 10–15 years of delay in the occurrence of visible aging signs among South African women, especially for wrinkles/texture and sagging, have to be compared to previous experiments,^18^, ^19^, ^20^, ^21^ where the impacts of sun‐exposures were observed later, that is, in the fifties and sixties, whereas Japanese and French women, using similar method, wrinkles/textures were already impacted in the forties. Hence, our findings suggest that a late onset of facial aging signs implies that related sun‐exposures manifestations subsequently arise later.

It was observed here that the changes in the signs of pigmentation changes with ages, and sun‐exposures were rather erratic. Such finding highlights the need to being more exhaustive, that is, to follow more specific markers in darkest skin tones for the reasons exposed above. Despite some limitations in the precision of changes in skin pigmentation, the present study nevertheless indicates that these are of very small amplitudes (close to zero) along the life span (Figure 2), inversely to other signs (wrinkles, sagging, cheek pores) that continuously increase by rather small steps between decades. In short, the facial skin aging of our studied cohort appears discrete and much more driven by skin surface events than its pigmentary aspect.

According to literature,^29^, ^30^, ^31^, ^32^, ^33^ if visible manifestations of aging or photo‐aging of dark‐skinned people are delayed versus other populations and especially in wrinkles/texture and sagging, the case of changes in the skin structure and its mechanical properties remains unknown. To such objective, coupling digital studies to video recordings of a standardized movement could probably be able to assess the structural alterations in dynamic behavior of facial skin occurring with aging.^34^

We acknowledge however that this study presents certain limitations since carried out outside of clinical facilities, without dermatologist‐supervised recruitment and on a relatively small cohort of South African women from the very same city (Pretoria). It however illustrates delayed manifestations of facial aging, similarly to previous studies carried out on Japanese subjects where no photo‐damages could be noticed at young ages. Nevertheless, such significant observations, mirroring dermatologists’ assessments, benefit from the accuracy, reliability and repeatability of the AI‐based automatic grading system as it was demonstrated weakly affected from lighting conditions, resolution of frontal cameras (if above five megapixels), or angle in the shooting.

With these new data and identified markers of aging and photo‐aging among South African women an evaluation more complete, objective with holistic appreciation of facial appearance and its variations^9^, ^10^ can be demonstrated and quantified. Indeed, our group reported that wrinkles/texture and sagging signs represent, in South African darker skin tones, 61% of the perception of age by a naïve panel. In fact, the need to reach a more inclusive and personalized experience in the skincare domain implies most accurate diagnostic and treatments in full transparency to the consumer. The new approach used here that leverages deep learning in dermo‐cosmetic is essential to increase our knowledge on all possible factors that modify facial skin on all skin types. Hence, such methodology obviously needs to be applied on subjects from many other geographical regions and different cultural features.

## CONFLICT OF INTEREST

All authors are employees of L'Oréal Research and Innovation Department and the Modiface company.
